# Impact of quadrivalent influenza vaccine on public health and influenza-related costs in Australia

**DOI:** 10.1186/s12889-016-3297-1

**Published:** 2016-07-22

**Authors:** Aurélien Jamotte, Chui Fung Chong, Andrew Manton, Bérengère Macabeo, Mondher Toumi

**Affiliations:** Creativ-Ceutical, 215 rue du Faubourg Saint Honoré, 75008 Paris, France; Sanofi Pasteur Asia Pacific, 38 Beach Rd, #18-11, South Beach Tower, Singapore, 189767 Singapore; Commercial Eyes, Level 11 500 Collins Street, Melbourne, VIC 3000 Australia; Sanofi Pasteur Global, 2, Avenue Pont Pasteur, 69007 Lyon, France; Public Health Laboratory, Faculty of Medicine, Aix-Marseille University, 27 boulevard Jean Moulin, 13385 Marseille Cedex 05, France; Present Address: Takeda Development Center Asia, Pte. Ltd, 21 Biopolis Road, Nucleos North Tower, Level 4, Singapore, 138567 Singapore

**Keywords:** Influenza, Vaccine, Quadrivalent, Australia, Cost, Benefit, Public health

## Abstract

**Background:**

Annual trivalent influenza vaccines (TIV) containing three influenza strains (A/H1N1, A/H3N2, and one B) have been recommended for the prevention of influenza. However, worldwide co-circulation of two distinct B lineages (Victoria and Yamagata) and difficulties in predicting which lineage will predominate each season have led to the development of quadrivalent influenza vaccines (QIV), which include both B lineages. Our analysis evaluates the public health benefit and associated influenza-related costs avoided which would have been obtained by using QIV rather than TIV in Australia over the period 2002–2012.

**Methods:**

A static model stratified by age group was used, focusing on people at increased risk of influenza as defined by the Australian vaccination recommendations. B-lineage cross-protection was accounted for. We calculated the potential impact of QIV compared with TIV over the seasons 2002–2012 (2009 pandemic year excluded) using Australian data on influenza circulation, vaccine coverage, hospitalisation and mortality rates as well as unit costs, and international data on vaccine effectiveness, influenza attack rate, GP consultation rate and working days lost. Third-party payer and societal influenza-related costs were estimated in 2014 Australian dollars. Sensitivity analyses were conducted.

**Results:**

Using QIV instead of TIV over the period 2002–2012 would have prevented an estimated 68,271 additional influenza cases, 47,537 GP consultations, 3,522 hospitalisations and 683 deaths in the population at risk of influenza. These results translate into influenza-related societal costs avoided of $46.5 million. The estimated impact of QIV was higher for young children and the elderly. The overall impact of QIV depended mainly on vaccine effectiveness and the influenza attack rate attributable to the mismatched B lineage.

**Conclusion:**

The broader protection offered by QIV would have reduced the number of influenza infections and its related complications, leading to substantial influenza-related costs avoided.

**Electronic supplementary material:**

The online version of this article (doi:10.1186/s12889-016-3297-1) contains supplementary material, which is available to authorized users.

## Background

Influenza is an acute infectious respiratory disease in humans, caused by influenza viruses A and B. In young children, the elderly, or persons with other serious chronic conditions, influenza can lead to complications of the underlying condition, pneumonia or even death [1]. Worldwide, influenza causes 3–5 million cases of severe illness and between 250,000–500,000 deaths every year [2]. In Australia, influenza has been estimated to cause on average 310,000 General Practitioner (GP) consultations and 18,400 hospitalisations annually between 1998 and 2005, leading to an annual expenditure of 115 million Australian dollars (A$) for the health care system [3].

Vaccination remains the most effective measure for preventing influenza and its complications [4, 5]. In Australia, influenza vaccination is recommended for everyone from 6 months of age [6], and is provided free under the National Immunisation Program to people at increased risk of influenza complications i.e. all adults aged 65 and older, Australian Aboriginal and Torres Strait Islander people aged between 6 months and 5 years or older than 15 years, pregnant women and all persons aged 6 months and older with specific comorbidities predisposing to severe influenza [7].

Immunisation against seasonal influenza using trivalent influenza vaccines (TIV) is currently recommended by public health policy makers around the world [8–10]. TIV contains strains of two influenza A sub-types (one each of H1N1 and H3N2), and one lineage of influenza B (Victoria or Yamagata), based on WHO recommendations [11].

However, over the decade 2000 through 2011 in Australia, the two distinct lineages of influenza B have been co-circulating at varying levels and with no regularity, resulting in mismatches between the circulating lineage and the vaccine in six of the 12 seasons [12]. More recently, during the early stages of the 2015 influenza season in New South Wales, 19 % of all influenza specimens tested positive were from the Victoria lineage, which was not included in TIV [13]. Two recent meta-analyses have demonstrated that the level of protection induced by TIV is sub-optimal when there is a mismatch between the circulating influenza B lineage and the actual B lineage included in the vaccine [14, 15].

Recently, quadrivalent influenza vaccines (QIV) including both B lineages in addition to both A subtypes have been developed in response to the evolution in influenza epidemiology. The potential impact of QIV in the US was estimated by Reed et al. [16], who considered a hypothetical scenario where QIV would have replaced TIV over ten influenza seasons. The study estimated that, within the seasons 2001–2002 to 2008–2009, QIV might have additionally prevented 340,000 influenza cases, 2,700 hospitalisations and 170 deaths per year compared with TIV. Another study by Lee et al. [17] expanded upon the results of Reed et al. to estimate the economic impact of QIV in the US and concluded that the use of QIV instead of TIV could substantially decrease influenza-related costs borne by society and health care payers.

In Australia, QIV was first approved by the Therapeutic Good Administration for the 2015 influenza season [18]. The objective of this study was therefore to estimate the additional benefit of using QIV rather than TIV, on influenza-related health and economic outcomes during the period 2002–2012 in Australia.

## Methods

Two different vaccination strategies were compared: the actual situation where TIV was administered over the period 2002–2012, and a second strategy where QIV would have been used instead of TIV during the same period.

### Model description

We used an age-stratified, static model. For each strategy, the number of influenza cases, GP consultations, working days lost, hospitalisations and deaths related to influenza, as well as associated costs, were estimated for each season over the period 2002–2012. Season 2009 was excluded from the scope due to the H1N1 pandemic, which rendered the year atypical and would have biased the estimated season-specific influenza attack rate. The potential public health and economic impact of QIV compared with TIV was captured as the difference in outcomes between the two strategies.

The expected influenza attack rate attributable to a specific virus strain $j$ ($j$ =A, B/Yamagata, B/Victoria) in a population partially vaccinated with a vaccine $i$ ($i$ =QIV, TIV) for a given year was computed as follows:

$$ A{R}_{i,j}=A{R}_{no\kern0.5em vac}\cdot \kern0.5em {p}_j\cdot \left(1-VC.V{E}_{i/j}\right) $$

Where ${AR}_{no\;vac}$ denotes the expected influenza attack rate without vaccination, ${p}_j$ the proportion of strain $j$, $VC$ the vaccine coverage rate and ${VE}_{i/j}$ the effectiveness of vaccine $i$ against strain $j$. The other influenza-related outcomes were assumed to be proportional to the number of influenza infections.

**Fig. 1:**
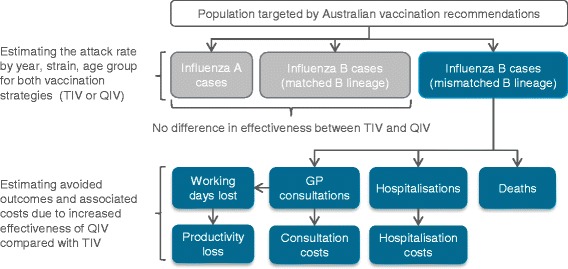
Model structure. Abbreviations: GP: general practitioner; TIV: trivalent influenza vaccine; QIV: quadrivalent influenza vaccine

To estimate the impact of QIV vs. TIV on resource use and influenza-related costs, the numbers of GP visits, hospitalisations and lost working days avoided were multiplied with corresponding unit costs. The economic impact was estimated by taking into account third-party payer (TPP) costs (GP consultations, hospitalisations), and societal costs (i.e. the sum of TPP costs and the loss of productivity due to work absenteeism), in 2014 Australian dollars ($). The model structure is presented in Fig. 1.

### Data inputs

#### Population

The population of interest was defined as the population eligible for free vaccination under the National Immunisation Program [7], including everyone older than 65 years and individuals aged 6 months to 64 years with at least one risk factor as defined in the Australian Immunisation Handbook [6], namely: pregnant women, persons with cardiovascular diseases, obesity, chronic respiratory conditions, chronic neurological conditions, metabolic diseases including diabetes mellitus, renal dysfunction, and immunocompromised conditions such as HIV and cancer.

To account for both local vaccination recommendations and the heterogeneity in the disease burden of influenza, the model was stratified into five age groups (6–59 months, 5–17 years, 18–49 years, 50–64 years, 65 years and older). For people aged from 6 months to 64 years, the analyses focused on people with risk factors.

To estimate the proportion of the general population with at least one risk factor, prevalence data were collected mainly from the 2012 Australian Health Survey [19], combined with other sources [20, 21], and were corrected to avoid double-counting when data were available. The population size (see Table 1) was assumed to be constant across the period, corresponding to the 2012 population estimates [22].

**Table 1:** Population, vaccine, average influenza-related outcome rates and economic inputs (in A$) by subgroup of population

Population subgroup	Population			Vaccine coverage rate [29, 30]	Vaccine effectiveness [31]			Average Influenza Attack rate [23–25]	GP consultations		Hospitalisations		Mortality rate^c^ [32, 33]	Productivity loss per GP consultation	
	Population size [22]	Proportion with RF [19–21]	Population at risk		Against influenza A	Against matched B lineage	TIV B lineage cross-protection, % matched B effectiveness		Rate^b^ [32]	Unit cost [34]	Rate^c^ [3]	Unit cost [35–37]		Working days lost [38–40]	Income loss [41]
Children with RF (6–59 months)	1,331,660	13.0 %	173,778^a^	41.3 %	59 %	66 %	67 %	18.8 %	91.0 %	$37	106	$3,944	0.6	0.64	$192
Children with RF (5–17 years)	3,673,678	13.9 %	509,239^a^	41.3 %	61 %	75 %	67 %	16.5 %	63.6 %	$37	16	$4,811	0.1	0.64	$192
Adults with RF (18–49 years	10,220,627	21.8 %	2,223,249^a^	36.2 %	61 %	77 %	68 %	3.6 %	62.6 %	$37	31	$5,644	0.3	2.59	$784
Adults with RF (50–64 years)	4,114,452	31.5 %	1,296,098^a^	36.2 %	61 %	73 %	67 %	3.6 %	62.6 %	$37	91	$7,497	4.1	2.59	$784
Elderly (65 years and older)	3,221,321	-	3,221,312	74.6 %	58 %	69 %	68 %	4.9 %	72.2 %	$37	440	$10,141	91.3	0^d^	$0^d^

*RF* risk factor, *TIV* trivalent influenza vaccine, *GP* general practitioner, ^a^Only people with risk factor; ^b^per influenza case; ^c^per 100,000 population; ^d^Elderly were assumed to be economically inactive

#### Influenza attack rate

Average annual attack rates in an unvaccinated population were taken from the pooled control arms of clinical trials presented in Cochrane reviews for healthy children, healthy adults and the elderly [23–25]. These rates were then distributed across the influenza seasons using the following formula:

$$ A{R}_{season, group}\kern0.5em =A{R}_{group}\times {V}_{season}/\left( Average\left({V}_{season}\right)\right) $$

where ${AR}_{season,\; group}$ denotes the age- and season-specific influenza attack rate, ${AR}_{group}$ the average attack rate for the given age group and ${V}_{season}$ the season-specific severity coefficient, defined as the year-specific number of influenza cases notified within the National Notifiable Diseases Surveillance System [26], adjusted by the number of yearly notifications from the other notified diseases to account for possible variations in the collection and testing capacities of the surveillance system.

#### Strain distribution

The distribution of influenza strains for the years 2002 to 2011 was estimated using data from the WHO Collaborating Centre for Reference and Research on Influenza in Melbourne, Australia [27]. Estimates for 2012 were derived from laboratory confirmed data from the Global Influenza Surveillance and Response System in Australia [28]. B lineages contained in the TIV formulation for Australia each year, corresponding to the southern hemisphere formulation, were retrieved from the World Health Organization website [11]. Influenza circulation by season is presented in Table 2.

**Table 2:** Influenza circulation and B lineage included in TIV by influenza season

Season	B lineage included in TIV [11]	Influenza circulation [27, 28]			B lineage mismatch^a^	Number of influenza notified cases (corrected with other disease notifications) [55]
		Influenza A (% of total)	Influenza B/Victoria (% of total)	Influenza B/Yamagata (% of total)		
2002	Yamagata	77.2 %	21.9 %	0.9 %	High	5,626
2003	Victoria	99.2 %	0.2 %	0.6 %	High	5,149
2004	Victoria	75.0 %	4.2 %	20.8 %	High	2,718
2005	Yamagata	78.5 %	10.4 %	11.1 %	Medium	5,541
2006	Victoria	65.6 %	32.9 %	1.5 %	Low	3,597
2007	Victoria	94.7 %	1.2 %	4.1 %	High	11,356
2008	Yamagata	36.7 %	32.4 %	30.9 %	Medium	8,882
2010	Victoria	87.3 %	11.5 %	1.2 %	Low	9,905
2011	Victoria	68.8 %	30.6 %	0.6 %	Low	18,658
2012	Victoria	69.5 %	4.3 %	26.3 %	High	32,274

*TIV* trivalent influenza vaccine, ^a^Low: <33 %, Medium: [33 %; 66 %], High: >66 %

#### Vaccine coverage and effectiveness

Coverage rates [29, 30], as well as strain-specific vaccine effectiveness, were assumed to be constant over time. The average effectiveness of TIV against matched and mismatched B lineages was estimated in a meta-analysis in which B lineage cross-protection was found to be approximately 68 % of the effectiveness against the matched B lineage in adults (i.e. vaccine efficacy was 77 % against matched B lineage and 52 % against mismatched B lineage) [14]. Vaccine effectiveness rates by strain and age group were sourced from Clements et al. [31] (see Table 1). Vaccine effectiveness against both B lineages for QIV was assumed to be the same as the vaccine effectiveness of TIV against the matched B lineage.

#### Resource use and economic inputs

The probability of an influenza infection requiring a GP consultation was derived from a US cost-effectiveness study in which the probability for high-risk patients was assumed to be twice that of low-risk patients [32]. Average hospitalisation rates were taken from Newall and Scuffham [3] in which excess hospitalisation (coded as influenza/pneumonia and other respiratory illness) rates by age group attributable to influenza in Australia were estimated using regression models based on data from 2000 to 2006. Mortality rates were based on the excess death (coded as respiratory or circulatory) rate presented in Newall et al. [33] for the elderly in Australia with an extrapolation to other age groups using the age-specific mortality rates of Molinari et al. [32].

The cost of a GP consultation was sourced from the Medical Benefits Schedule [34] while hospitalisation costs were estimated using reported costs from Diagnosis-Related Groups for respiratory conditions in the National Hospital Cost Data Collection Report 2011–2012 [35]. Costs were adjusted by age group using the average length of stay from the Australian-refined diagnosis-related groups data cubes [36] and inflated to 2014 rates [37].

The numbers of working days lost per GP consultation for adults and young children were respectively taken from a French study [38] reporting an average of 4 working days lost, and from an Italian study [39] in which parental working days lost were estimated to be 0.98 days. These estimates were then adjusted with the Australian labour force participation rate (64.8 %) [40] to obtain the number of working days lost per GP consultation. The cost of a working day lost was assumed to be the median Australian daily wage for full-time employees [41].

The cost of vaccination was not taken into account in this analysis which is aimed at measuring the impact of QIV on influenza burden, while assuming price parity between QIV and TIV.

### Sensitivity analyses

Sensitivity analyses were conducted to test the impact of modelling assumptions and the uncertainty around inputs.

First, we conducted two scenario analyses in which we tested different assumptions for the model. In a first scenario analysis (scenario 1), influenza attack rates were assumed to be constant over the study period to test the impact of the distribution of attack rates using the number of notified cases as a proxy of the season severity. In a second scenario analysis (scenario 2), hospitalisation and death rates in high-risk patients were adjusted using ratios between high-risk patients and the general population [42–44].

Second, we performed a univariate deterministic sensitivity analysis to quantify the effects of the uncertainty around data inputs on the number of avoided cases and total societal costs avoided, in which each group of parameters was varied one at a time to the lower and upper bounds of the range of estimated values. Lower and higher bound values used in the deterministic sensitivity analyses were taken from published literature and informed assumptions, and are presented in Additional file 1.

## Results

### Base case analysis

Over the period 2002–2012, it was estimated that substituting QIV for TIV would have reduced the number of influenza cases by over 68,000 in addition to the cases avoided with TIV, leading to 47,500 GP consultations, 3,500 hospitalisations and 680 deaths avoided in Australia (see Table 3). The majority (58.4 %) of influenza cases would have been avoided in 2012 - a season with a high attack rate, a relatively high B strain circulation (30.5 %) and a very high B lineage mismatch (86.8 %). Seasons 2002 and 2008 were also years associated with a substantial impact of QIV, with around 5,800 and 13,500 influenza infections additionally avoided, respectively. The avoided resource use was associated with a reduction of influenza-related TPP costs of approximately A$ 36.5 million over the period 2002–2012 compared with the TIV strategy. Avoided hospitalisation costs represented 95 % of total influenza-related TPP costs avoided. Productivity loss avoided was estimated at A$ 10.0 million, representing 21.5 % of the A$ 46.5 million of additional societal costs avoided. On average, over the five targeted population subgroups, QIV was estimated to additionally prevent 92 influenza cases per 100,000 person-years, leading to avoided influenza-related societal costs of A$ 62,700 per 100,000 person-years.

**Table 3:** Number of avoided outcomes and associated cost offsets (in A$) for total recommended population (*n* = 7,423,675) by season

	Outcomes avoided										Influenza-related cost offsets			
	Influenza cases		GP consultations		Working days lost		Hospitalisations		Deaths		Total TPP		Total Societal	
	Total	Rate per 100,000 population	Total	Rate per 100,000 population	Total	Rate per 100,000 population	Total	Rate per 100,000 population	Total	Rate per 100,000 population	Total	Rate per 100,000 population	Total	Rate per 100,000 population
2002	5,793	78	4,034	54	2,808	38	299	4.0	58	0.78	$3,097,560	$41,725	$3,946,994	$53,168
2003	155	2	108	1	75	1	8	0.1	2	0.02	$82,892	$1,117	$105,623	$1,423
2004	2,663	36	1,854	25	1,291	17	137	1.9	27	0.36	$1,423,708	$19,178	$1,814,127	$24,437
2005	2,707	36	1,885	25	1,312	18	140	1.9	27	0.36	$1,447,482	$19,498	$1,844,420	$24,845
2006	262	4	182	2	127	2	14	0.2	3	0.04	$140,054	$1,887	$178,461	$2,404
2007	2,209	30	1,538	21	1,070	14	114	1.5	22	0.30	$1,180,882	$15,907	$1,504,711	$20,269
2008	13,543	182	9,430	127	6,564	88	699	9.4	135	1.82	$7,241,013	$97,539	$9,226,692	$124,287
2010	544	7	379	5	264	4	28	0.4	5	0.07	$291,114	$3,921	$370,945	$4,997
2011	493	7	343	5	239	3	25	0.3	5	0.07	$263,571	$3,550	$335,849	$4,524
2012	39,902	538	27,784	374	19,341	261	2,058	27.7	399	5.38	$21,334,862	$287,389	$27,185,450	$366,199
Total	68,271	920	47,537	640	33,091	446	3,522	47.4	683	9.20	$36,503,138	$491,712	$46,513,271	$626,553

*GP* general practitioner, *TPP* third-party payer

The overall impact of QIV was highest for the elderly with 36,322 additional cases avoided versus 15,977 for children and 15,971 for adults below 65 years of age (see GP: general practitioner; TPP: third-party payer Table 4). The elderly population accounted for 73 % of the avoided influenza-related societal costs (A$ 34.0 million of A$ 46.5 million for the entire population of interest). Relative to population size, the impact of QIV in terms of influenza cases and GP consultations avoided was greatest for young children, with an average of 239 influenza cases avoided per 100,000 person-years, leading to 217 GP consultations avoided. However, QIV was most beneficial for the elderly in terms of hospitalisation and mortality, with 10.1 additional hospitalisations and 2.10 additional deaths avoided per 100,000 person-years. The economic impact of QIV was also highest for the elderly, with mean influenza-related societal costs avoided of A$ 105,560 per 100,000 person-years, the results being driven by hospitalisation costs.

**Table 4:** Number of avoided outcomes and associated cost offsets (in A$) over 2002–2012 (2009 excluded) by population subgroup

	Children with RF 6–59 months (*n* = 173,778)		Children with RF 5–17 years (*n* = 509,239)		Adults with RF 18–49 years (*n* = 2,223,249)		Adults with RF 50–64 years (*n* = 1,296,098)		Elderly 65 years and older (*n* = 3,221,312)		Total population (*n* = 7,423,675)	
	Total over the period	Annual average^a^	Total over the period	Annual average^a^	Total over the period	Annual average^a^	Total over the period	Annual average^a^	Total over the period	Annual average^a^	Total over the period	Annual average^a^
Outcomes avoided												
Influenza cases	4,153	239	11,824	232	10,240	46	5,731	44	36,322	113	68,271	92
GP consultations	3,780	217	7,520	148	6,410	29	3,588	28	26,239	81	47,537	64
Working days lost	2,400	138	4,776	94	16,616	75	9,299	72	0^b^	0^b^	33,091	45
Hospitalisations	23	1.3	11	0.2	86	0.4	143	1.1	3,257	10.1	3,522	4.7
Deaths	0	0.01	0	0.00	1	0.00	6	0.05	675	2.10	683	0.92
Influenza-related cost offsets												
GP consultations	$140,034	$8,058	$278,618	$5,471	$237,508	$1,068	$132,923	$1,026	$972,167	$3,018	$1,761,250	$2,372
Hospitalisations	$92,186	$5,305	$54,790	$1,076	$487,832	$2,194	$1,075,050	$8,295	$33,032,031	$102,542	$34,741,889	$46,799
Income loss	$726,069	$41,781	$1,444,619	$28,368	$5,026,395	$22,608	$2,813,051	$21,704	$0^b^	$0^b^	$10,010,133	$13,484
Total TPP	$232,220	$13,363	$333,408	$6,547	$725,340	$3,263	$1,207,973	$9,320	$34,004,198	$105,560	$36,503,138	$49,171
Total Societal	$958,288	$55,145	$1,778,027	$34,915	$5,751,735	$25,871	$4,021,024	$31,024	$34,004,198	$105,560	$46,513,271	$62,655

*RF* risk factor, *GP* General Practitioner, *TPP* Third-Party Payer^a^ Per 100,000 person-years; ^b^Elderly were assumed to be economically inactive

Finally, considering all influenza cases (A and B), it was estimated that substituting QIV for TIV would have reduced the number of influenza cases by 1.02 % on average over the period 2002–2012 (2009 excluded). The reduction ranged from 0.05 % in 2003 to 2.36 % in 2008, with a reduction by 1.90 % in 2012, year with the most influenza burden avoided (39,900 cases) with QIV compared to TIV.

### Sensitivity analyses

Results of the scenario analyses conducted showed limited variations in outcomes (see Table 5). In scenario 1, assuming constant attack rates for influenza over the study period would lead to an overall reduction of 14 % of QIV benefits compared to base case. When considering an increased risk of hospitalisation and deaths for people with risk factor (scenario 2), the number of hospitalisations and deaths avoided increased by 16 and 1 % respectively while the total influenza-related societal costs avoided increased by 8 % compared to base case.

**Table 5:** Results of scenario analyses over 2002–2012 (2009 excluded) for total recommended population (*n* = 7,423,675)

	Total over the period (% variation compared to base case)		
	Base case	Scenario 1	Scenario 2
Outcomes avoided			
Influenza cases	68,271	58,468 (−14 %)	68,271 (0 %)
GP consultations	47,537	40,711 (−14 %)	47,537 (0 %)
Working days lost	33,091	28,339 (−14 %)	33,091 (0 %)
Hospitalisations	3,522	3,016 (−14 %)	4,095 (16 %)
Deaths	683	585 (−14 %)	693 (1 %)
Influenza-related cost offsets (in A$)			
GP consultations	$1,761,250	$1,508,357 (−14 %)	$1,761,250 (0 %)
Hospitalisations	$34,741,889	$29,750,615 (−14 %)	$38,300,256 (10 %)
Income loss	$10,010,133	$8,572,811 (−14 %)	$10,010,133 (0 %)
Total societal costs	$46,513,271	$39,831,784 (−14 %)	$50,071,639 (8 %)

*GP* general practitioner, Scenario 1: Constant attack rate over study period; Scenario 2: Increased risk of hospitalisation and death for population with risk factor

**Fig. 2:**
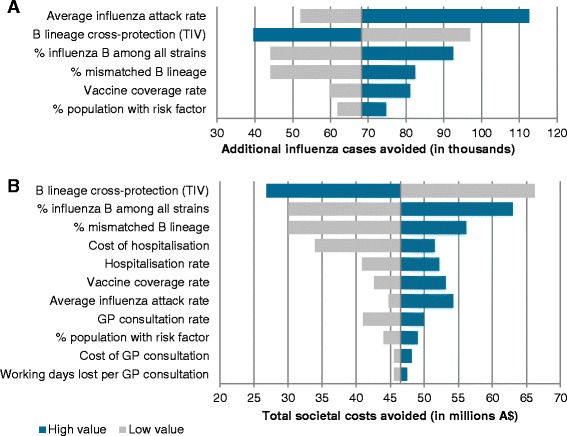
Deterministic sensitivity analyses on total influenza cases avoided (**a**) and societal cost offsets (**b**) over the period 2002–2012 (2009 excluded). High value: results of the model when the parameter value is set to the lower bound of the parameter range. Low value: results of the model when the parameter value is set to the upper bound of the parameter range. Example of interpretation for Fig. 2b: In the base case, QIV was estimated to lead to the avoidance of $46.5 million in societal costs. When considering the lower and upper bounds of the estimated degree of B lineage cross-protection, cost offsets were estimated to be A$ 66.2 million and A$ 26.8 million respectively. Lower and higher bounds used in the deterministic sensitivity analyses are available in Additional file 1. Abbreviations: GP: general practitioner; TIV: trivalent influenza vaccine

Deterministic sensitivity analyses conducted for several inputs on the number of influenza cases avoided and societal costs avoided showed that the variables with the most impact on both outcomes were the vaccine effectiveness against mismatched B, the proportion of B strain and the proportion of mismatched B lineage (see Fig. 2). Age-specific influenza attack rates over the period was the parameter with the largest impact on the number of influenza cases avoided, estimated to be from 52,000 to 113,600 cases when considering the lower and higher estimates, respectively.

## Discussion

In recent years, the interest in QIV has grown mainly because TIV only matches the predominant circulating B lineage, and predicting which of the two B lineages will circulate in any given season remains a challenge. We modelled the impact of QIV compared to TIV vaccination in Australia for five population groups at increased risk of influenza complications over 10 seasons, based on the same approach as in Reed et al. [16].

Overall, the use of QIV instead of TIV would have substantially reduced the clinical burden of influenza while avoiding thousands of hospitalisations and hundreds of deaths associated with influenza over the 10 seasons in the population at risk of influenza. The overall impact of QIV was highest for the elderly who contributed the most to additional deaths avoided over the period. Furthermore, assuming no increase in vaccination costs, the use of QIV instead of TIV would have avoided A$ 46.5 million in influenza-related societal costs over 10 years. The analyses suggested that cost offsets would be higher in the elderly, being essentially driven by hospitalisations, than in the other targeted groups. The impact of QIV mainly depends on influenza B strain circulation, and influenza B co-circulation of B/Yamagata and B/Victoria is expected to continue in upcoming influenza seasons [45].

Compared to the US study by Reed et al. [16], two enhancements to the model were made, leading to more accurate estimates of the public health impact and influenza-related costs avoided. First of all, our analysis accounted for TIV cross-protection against mismatched B lineage. Secondly, the population was stratified to account for differences in vaccination coverage, vaccine effectiveness and risks of complications between groups. These improvements to the model, along with influenza circulation informed by data specific to Australia, allowed us to provide more realistic estimates of the benefits of QIV.

Contrary to our study, the study by Lee et al. [17] included lifetime productivity loss in the event of death in societal costs to assess the economic impact of QIV based on the model by Reed et al. Influenza-related societal costs avoided estimated by Lee et al. were substantially higher than those estimated in our analysis: about US$ 110,000 per 100,000 persons in the general US population, compared to approximately US$ 46,000 (US$ 1 = A$ 1.6). Clements et al. [31] estimated influenza-related costs avoided associated with the use of QIV in the US at US$ 41,000 (without vaccination cost and lifetime productivity loss) for 100,000 people accounting for cross-protection and using stratification.

In Australia, Newall and Scuffham [3] estimated that around 310,000 GP visits and 18,400 hospitalisations annually were due to influenza in the general population over the period 1998–2005. In our study, when considering the entire Australian population, the model estimated the number of hospitalisations under TIV at 14,700 annually across targeted groups while the estimated number of GP consultations per year was substantially higher with 436,000 GP consultations per year. Recently, Milne et al. [46] estimated, using a dynamic transmission model, that QIV would have reduced hospitalisations and deaths by 2.2 and 2.1 % respectively, compared with TIV over 2001–2011 in Albany, Western Australia. Our model however, estimated the reduction to be 2.4 and 3.7 % respectively, over 2002–2012. These slightly higher rates can be explained by a slightly different coverage rate considered in our model (17.5 vs. 15 %) combined with the inclusion of 2012, a year with high B mismatch.

There are limitations in the available data, common to most influenza simulation models [47], which affected our analysis. To address the impact of the following limitations, we conducted a number of scenario analyses and sensitivity analyses.

Firstly, in the absence of reliable data to estimate the annual incidence of influenza across age groups for a given country and period in time, extrapolations were made in order to derive season-specific incidences over 2002–2012. Because the randomized clinical trials considered in the calculations of average age-specific influenza attack rates were not specific to Australia and to the period of analysis, and because the distribution of these average rates relied on local surveillance data which might be biased due to changes in the surveillance networks over the years, the obtained seasonal incidences could very well be different from what really happened in Australia over the period 2002–2012.

Secondly, as no Australian data were available, probabilities of GP consultation following influenza infection were taken from a US study, while the number of workdays lost per influenza infection were derived from European literature. These estimates are likely to be underestimated since Australia ranks well internationally in terms of health care accessibility, with nearly 85 % of Australians consulting a GP at least once a year as against less than 70 % in the US [48], and also in terms of social security in general. Moreover, the range of values tested in the sensitivity analyses were sufficiently wide to cover the range of estimates reported in the literature for developed countries, such as the GP consultation rates considered in Preaud et al. [49] for people with risk factors in Europe (32–70 %) and the range of the mean number of working days lost per episode following physician diagnosis of influenza (3.7–5.9 days) as reported in an international review [50]. Sensitivity analyses supported the fact that these parameters did not have a critical impact on the model outcomes, with a maximum of 12 % variability around base case values of societal costs avoided with QIV.

People with risk factors are known to be at increased risk of influenza complications, but there were no robust data available to estimate the influenza burden for such people. The main analysis, in which we considered estimates for the general population across all age groups, is likely to underestimate the impact of QIV in people aged 6 months to 64 years with risk factors. We ran a scenario analysis to provide an estimate of this underestimation, while also assuming the same influenza strain distribution for all age groups. If strain distributions specific to each age group would have been available, the estimated impact of QIV may have been slightly lower since the majority of hospitalisations and deaths related to influenza occur in the elderly and in very young children (<6 months) [3], the latter group not being directly eligible for influenza vaccination. Indeed, limited evidence showed that although influenza B causes disease in all age groups, its incidence relative to influenza A appears to be highest among older children and young adults [51, 52]. Finally, resource use was assumed to be similar for all influenza types, supported by the findings of a recent prospective study which found no difference in clinical features between cases due to influenza type A and type B over four seasons [53].

Overall, the influenza-related costs avoided, estimated by the model are likely to be conservative as they do not reflect the entire economic burden avoided in Australia. Medical costs of death, medication costs or laboratory tests, transportation costs as well as potential higher costs for care delivered in a private setting were omitted, indirect costs did not include societal costs of premature death and the loss of working days due to hospitalisations, and furthermore, the analysis only accounted for part of the Australian population.

Finally, some additional analyses and improvements of the model should be considered in future research. Firstly, vaccination costs that were not taken into account in our study as we mainly focused on the medical costs and loss of productivity related to influenza could be included, following which a cost-effectiveness analysis could be conducted taking into consideration the vaccination costs associated with QIV and TIV. Secondly, because of the scarcity of local data, we used a static model to estimate the impact of QIV. Static models are unable to account for changes in the force of infection arising from the reduction in the prevalence of infectious individuals that can be brought about by vaccination or acquired immunity [54]. These models are only able to capture the impact of direct protection at the very start of an influenza season, resulting potentially in the underestimation of the benefits of vaccination compared to dynamic models. In addition, static models do not take into account different contact rates between individuals according to their age or social characteristics, which have an impact on the transmission of influenza strains across population groups. However, as dynamic modelling is a complex approach which requires extensive data, subject to data availability, a further step could be to refine the estimation of QIV benefits using a dynamic model.

## Conclusions

In Australia, the use of QIV instead of TIV over the period 2002–2012 could have led to significant reductions in the number of influenza infections and its related complications, leading to the avoidance of influenza-related costs. Sensitivity analyses demonstrated that the results are mainly driven by the characteristics of influenza circulation and the estimated vaccine effectiveness. The use of QIV instead of TIV is expected to prevent much influenza-related burden in years with high B circulation and mismatch.

## Abbreviations

A$, Australian dollar; GP, general practitioner; QIV, quadrivalent influenza vaccine; TIV, trivalent influenza vaccine; TPP, third-party payer

## Additional files

Additional file 1: Input parameters for the deterministic sensitivity analyses. The table contains the low and high values for every parameter which was tested in the deterministic sensitivity analyses as well as the sources for these values. (XLSX 15 kb)
